# Establishing hierarchy: the chain of events leading to the formation of silicalite-1 nanosheets†

**DOI:** 10.1039/c6sc01295g

**Published:** 2016-06-22

**Authors:** Xiaochun Zhu, Maarten G. Goesten, Arjan J. J. Koekkoek, Brahim Mezari, Nikolay Kosinov, Georgy Filonenko, Heiner Friedrich, Roderigh Rohling, Bartłomiej M. Szyja, Jorge Gascon, Freek Kapteijn, Emiel J. M. Hensen

**Affiliations:** a Eindhoven University of Technology, Department of Chemical Engineering and Chemistry, Schuit Institute of Catalysis, Inorganic Materials Chemistry Netherlands e.j.m.hensen@tue.nl; b Eindhoven University of Technology, Department of Chemical Engineering and Chemistry, Laboratory of Materials and Interface Chemistry and TU/e Center of Multiscale Electron Microscopy Netherlands; c Delft University of Technology, Chemical Engineering Netherlands

## Abstract

In applying a multi-scale spectroscopic and computational approach, we demonstrate that the synthesis of stacked zeolite silicalite-1 nanosheets, in the presence of a long-tail diquaternary ammonium salt surfactant, proceeds through a pre-organised phase in the condensed state. *In situ* small-angle X-ray scattering, coupled to paracrystalline theory, and backed by electron microscopy, shows that this phase establishes its meso-scale order within the first five hours of hydrothermal synthesis. *Quasi in situ* vibrational and solid-state NMR spectroscopy reveal that this meso-shaped architecture already contains some elementary zeolitic features. The key to this coupled organisation at both micro- and meso-scale, is a structure-directing agent that is ambifunctional in shaping silica at the meso-scale whilst involved in molecular recognition at the micro-scale. The latter feature is particularly important and requires the structure-directing agent to reside within the silica matrix already at early stages of the synthesis. From here, molecular recognition directs stabilization of precursor species and their specific embedding into a lattice, as shown by force-field molecular dynamics calculations. These calculations, in line with experiment, further show how it is possible to subtly tune both the zeolite topology and aspect ratio of the condensating crystals, by modifying the headgroup of the structure-directing agent.

## Abstract 2

In applying a multi-scale spectroscopic and computational approach, we demonstrate that the synthesis of stacked zeolite silicalite-1 nanosheets, in the presence of a long-tail diquaternary ammonium salt surfactant, proceeds through a pre-organised phase in the condensed state.

## Graphical abstract

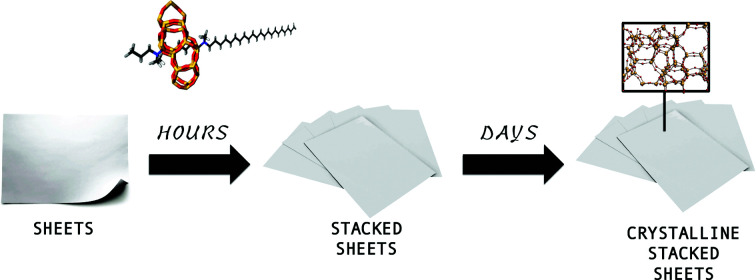

## Introduction

Zeolites are crystalline, porous silicates of great importance to catalysis, adsorption and separation.^1,2^ The appeal of zeolites is attributable to distinct pore dimensions, high surface areas, outstanding chemical and thermal stabilities, and the availability of more than 220 topologies that can be targeted by appropriate synthesis. This synthesis usually involves the judicious choice of a structure-directing agent (SDA) that induces specific molecular interaction with the condensating silica scaffold.

Catalytic application usually features zeolites where some four-coordinate silicon sites have been substituted by four-coordinate aluminium. In this way, aluminium-containing zeolites contain inherently acidic hydroxyl groups that charge-balance the inorganic framework. These acidic sites are located in intersecting channels and cavities of micropore dimensions, adding confinement, and rendering zeolites efficient acid-catalysts for shape-selective hydrocarbon conversion.^3,4^ Confinement has disadvantages too: it invokes diffusional limitations for products and reactants, which may seriously limit the catalytic potential of zeolites. As zeolite crystal dimensions are usually much larger than the micropores,^5^ a large fraction of the internal acid sites remains unused during conversion,^6^ resulting in lower rates and undesired side-reactions, such as coking. Thus, one of the grand synthetic challenges in materials chemistry is to fabricate zeolites that do not suffer from mass transport limitations, whilst retaining confinement, so valuable in shape-selective conversion.

Herein, several strategies have been developed, such as the synthesis of nano-scaled crystals, and the introduction of mesoporosity by post-synthetic leaching.^7–10^ The latter protocol matters the creation of a so-called *hierarchical zeolite*, with a hierarchical arrangement of two types of pore size, usually micro- and mesopores. Such zeolites have indeed shown to possess improved molecular transport due to the presence mesopores, and at no cost of shape-selectivity in catalysis.^11,12^

Yet, for the sake of control, and in context of human's long-standing efforts in *designing* new materials, it is very appealing to directly craft such hierarchical architectures in a one-step synthesis.^13–17^ To achieve this, the focus has been on SDAs that direct structure formation at both micro- and meso-scale. As has turned out, the realisation of tailored multi-scale synthesis is a formidable challenge in itself, much due to undesired synergy between structure direction at the micropore and mesopore scale level. A typical example is the synthesis of MCM-41, which is synthesized with cetyltrimethylammonium bromide (CTAB) – a long-tail analogue of typical SDAs that can also promote zeolite formation. Although hexagonally *shaped* at the meso-scale, MCM-41 does not contain order at the molecular level and lacks the acidity that is inherent to crystalline aluminium-bearing silica frameworks.^18,19^

**Fig. 1:**
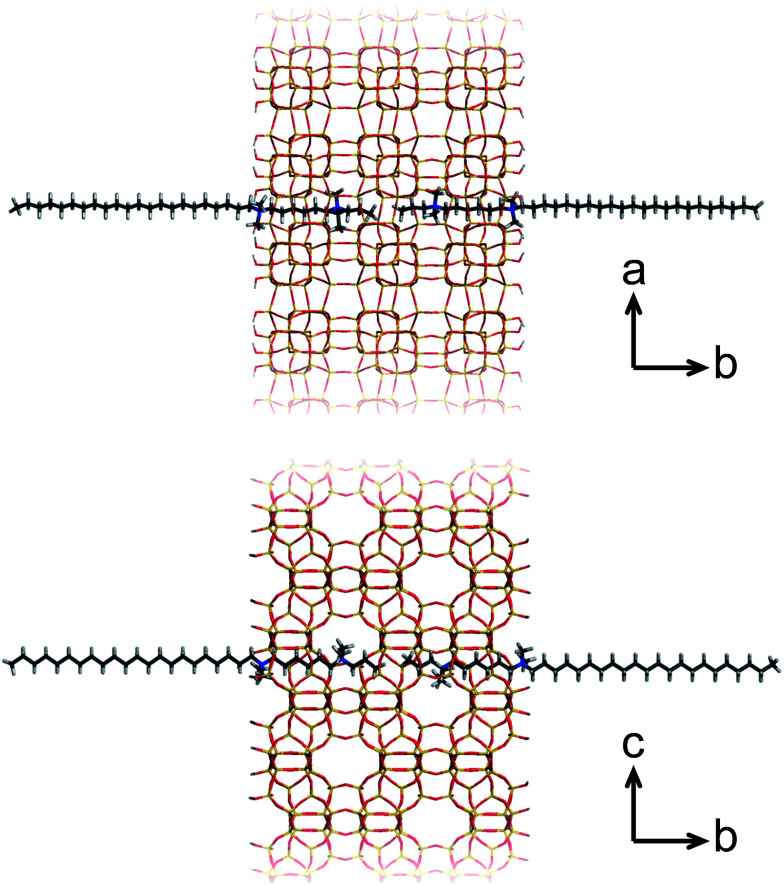
The silicalite-1 framework templated by C_22-6-3_, viewed in two directions.

A more recent, successful approach involves the synthesis of ultrathin zeolite sheets that stack through physical forces as a hierarchical array. Herein, diquaternary ammonium salt (DQAS) SDAs of the general type C_*i*_H_2*i*+1_-N^+^(CH_3_)_2_–C_*j*_H_2*j*_-N^+^(CH_3_)_2_–C_*k*_H_2*k*+1_, abbreviated C_*i-j-k*_, have proven a major step forward. These SDAs usually come in the form of C_22-6-6_ or C_22-6-3_ (the latter leading to materials of slightly higher crystallinity)^20^ and entirely fulfill the requirement of aforementioned structure direction at both micro- and mesoscale; the diquaternary headgroups promote formation of layers of the microcrystalline MFI topology, while the alkyl tails give rise to hydrophobic domains in between these layers, *i.e.*, they give rise to a stacked nanosheet architecture (Fig. 1). The MFI-topologic nanosheets can be synthesized in all-silica (silicalite-1) or aluminium-containing (ZSM-5) form. The latter species – upon removal of the SDA – were shown to act as highly efficient and long-lasting catalysts in a variety of catalytic reactions of industrial relevance.^11,12,15^

It is fair to state that the development of DQAS templates to direct formation of stacked-sheet architectures matters one of the bigger breakthroughs in zeolite chemistry in the current century. That stated, it did not come with commensurate understanding from the all-important perspective of solid-state synthesis. A quintessential question standing central towards a general approach in one-step hierarchical zeolite synthesis – *should one “meso-shape” condensed silica before crystallisation, or create mesoscale organisation with already crystalline structures?* – has not been answered.

To make matters more complicated, in resolving above question, one does not escape from involving an overlying discussion that has been holding those that study the process of zeolite crystallization, in its grip. In essence, there exist two general, opposing views on zeolite formation and the related role of the SDA. At first, there is a *classical view*, in which formation of the zeolite lattice is considered to start by spontaneous nucleation and ensuing addition of molecules, during which the topology of the growing matrix is directed by the SDA.^21–23^ Mostly on the basis of advanced (*quasi*) *in situ* characterisation a second, non-classical view turned up, in which it was postulated that pre-organized, supramolecular precursor building blocks are stabilized by the SDA, and further self-assemble towards the zeolite framework.^24–30^ Within this view, specific silica–SDA interactions determine the structure and connectivity of such precursor building blocks, and regulate the final topology by reticular pathways, not unlike those encountered in Metal–Organic Framework (MOF) crystallization.

A twist to the tale of these opposing views was recently provided by an *in situ* imaging approach, which convincingly demonstrated that during crystallisation of silicalite-1 – a popular case study – *both* the classical and non-classical mechanisms occur. In this scenario, crystallisation commences by precursor self-assembly, after which structural rearrangement and 3D lattice evolution occurs by accretion of silica molecules.^31^

In addition to its general significance, this bridging of theories confirms the prowess the SDA should have in stabilizing precursor species to initiate crystallisation. Referring back to the synthesis of hierarchical zeolites, this translates to two-fold structure direction at both micro- (0.1–2 nm) and meso- (2–50 nm) length scales.

If we remain with the synthesis of silicalite-1, and then investigate its recent appearance as a hierarchically stacked entity at the nanoscale, how is structure direction at both the supramolecular and colloidal scales established and commingled? That is the main question of the current case study, in which we build on aforementioned knowledge, and extend it to *meso-shaped*, crystalline zeolites.

We apply a multi-scale approach to the synthesis of stacked silicalite-1 nanosheets by C_22-6-3_. Our ensuing analysis is split up in two parts. At first, we will probe the colloidal length scale by *in situ* synchrotron Small-Angle X-ray Scattering (SAXS), backed by electron microscopy (EM). We then move to the supramolecular length scale, where vibrational spectroscopy, solid-state NMR and high-level molecular simulations reveal molecular order and local SDA–silica interactions.

It will become apparent that, from the earliest of synthesis times, silica is shaped towards stacked, sheet-like entities, which progressively arrange themselves towards meso-shaped arrays. At similarly early time scales, and at the molecular level, this inorganic–organic precursor phase already contains some of the structural features that are distinctive of the crystalline zeolite.

## Results and discussion

### The meso scale: X-ray scattering and electron microscopy

For the SAXS experiments, we used an in-house developed synchrotron cell, in which the hydrothermal synthesis of the nanosheet stacks could be followed *in situ*. This cell contains a rotating chamber in order to prevent sedimentation from happening.^32^

**Fig. 2:**
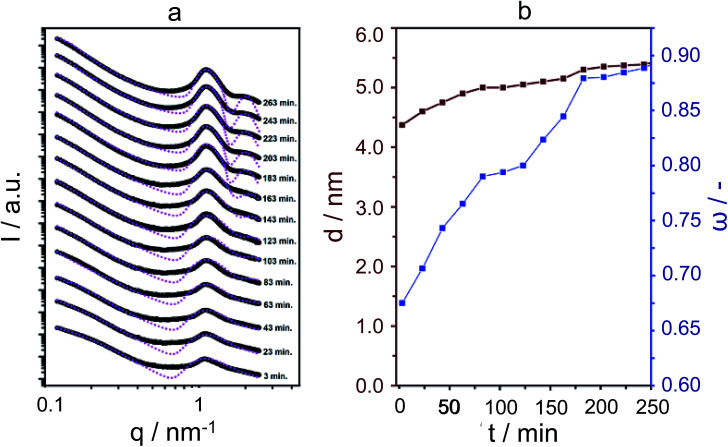
*In situ* SAXS patterns of silicalite-1 nanosheets synthesis at 135 °C using C_22-6-3_, with time intervals of 20 minutes and corresponding fittings using the paracrystalline structure factor, which are displayed in pink (a). Simulations of the scattering intensity predicted by the model showing dependence on stacking distance *d* and order *ω* (b).

**Fig. 3:**
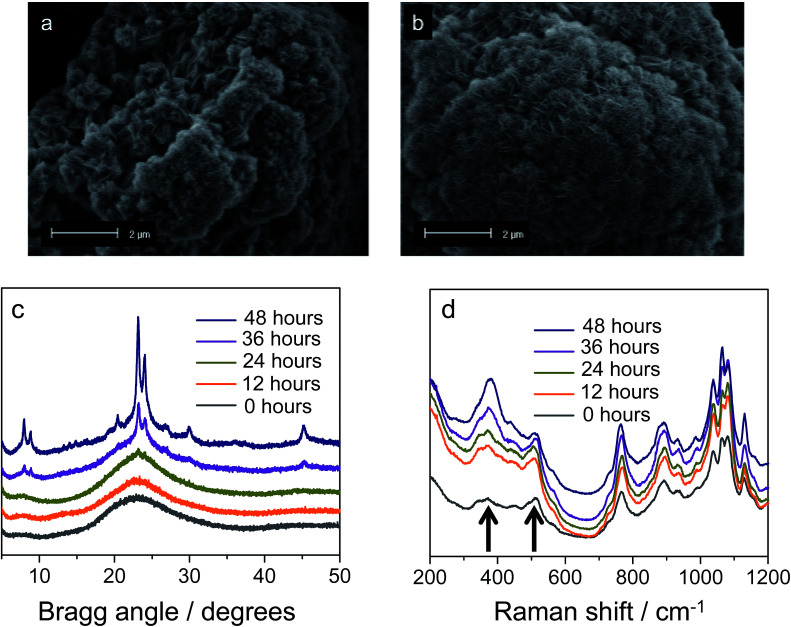
Morphology of silicalite-1 nanosheets synthesized with DQAS C_22-6-3_. SEM images of freeze-dried samples obtained after aging the C_22-6-3_-silica gel at room temperature (a) and then hydrothermal synthesis for 72 h (b). (c) XRD patterns and (d) Raman spectra of freeze-dried silicalite-1 nanosheets as a function of synthesis.

Fig. 2a displays the SAXS patterns obtained in hydrothermal synthesis, with time intervals of 20 minutes. It is clear, and remarkably so, that first- and second-order *quasi*-Bragg peaks, at 1 nm^−1^ and 2 nm^−1^, exist and develop during the very early times of hydrothermal synthesis. Such *quasi*-Bragg peaks are typically observed for stacked materials and find their origin in translational symmetry in the stacking direction.^33,34^ This observation of early sheet-like entities was confirmed by both scanning and transmission electron microscopy (Fig. 3a and ESI Fig. 4†).

The *quasi*-Bragg peaks were fitted with the characteristic representation *I*(*q*) = *P*(*q*)*S*(*q*). Here, *P*(*q*) is the form factor, responsible for single-entity scattering, and *S*(*q*), the structure factor, which describes the interference caused by inter-particle scattering. For *P*(*q*), a function derived for sheet-like scatterers is employed, and for *S*(*q*), paracrystalline theory (PT), as developed by Hosemann.^35^ The latter model is of special significance to our system, as we can obtain information on stacking disorder. Whereas it is expected that the position of the first- and second-order *quasi*-Bragg peaks in reciprocal *q*-space depends on the distance between sheets, *d* (by 2π/*q*), PT allows for analysing the line-shape to obtain information on stacking disorder *δ*, defined as a standard deviation in stacking distance. The fittings are shown in Fig. 2, using *I*(*q*) = *P*(*q*)*S*(*q*). Overall, the model was able to fit the *quasi*-Bragg peaks well, with a goodness of fit exceeding 90% for all cases. It might be noted that the model incorrectly predicts steep minima next to the peaks, but we underline that this is a typical observation in modelling SAXS data; models are derived for scattering entities in vacuum, and in solution-state reality, one observes typical smoothening of the troughs predicted by the mathematical model.^34^ Our focus lied on obtaining information on stacking distance and disorder from fitting the *quasi*-Bragg peak position and line-shape; the model performed herein very well.

In defining a more intuitive parameter for order *ω*, rather than disorder, we set *ω* = 1 − *δ*/*d*, where *ω* = 1 represents perfect stacking and *ω* = 0 total absence of such order. Corresponding evolutions of *d* and *ω* during hydrothermal synthesis are shown in Fig. 2b. Here, it is clear that sheets arrange rapidly into an ordered structure during the first hours of the synthesis, whilst the interlayer distance increases subtly. The pattern obtained after 263 minutes is essentially identical to that after 12 hours of synthesis. The same picture arises from SEM, which indicates that the meso-scale structure of the freeze-dried sample at time-zero remains preserved over longer periods of heating (72 hours, Fig. 3a and b) and strongly resembles the globular zeolite particles comprised of stacked sheets in the fully crystallized zeolite (ESI Fig. 1a†).

It is important to stress that during a 12 hour X-ray scattering experiment, no crystalline order was observed by the wide-angle camera. Further consistent with these observations are the *quasi in situ* XRD patterns of freeze-dried samples after 12 h of hydrothermal synthesis at 135 °C: these did not contain any indication for long-range atomic ordering typical for MFI-topologic zeolites (Fig. 3c). In fact, the earliest onset of crystallinity appears after 24 h, which then develops into a typical silicalite-1 nanosheet pattern during the following 48 hours (*t* = 24–72 h).

Thus, it appears that the meso-scale architecture is established at very early times of synthesis, at least within the first five hours of hydrothermal heating, after which bulk crystallization, *i.e.*, organisation at the molecular scale, occurs.

### The micro scale: vibrational spectroscopy, solid-state NMR and molecular simulations

Our analysis moves to the molecular scale. Raman scattering is very sensitive to the detection of *zeolitic* features, which may or may not be present in materials that do not yet contain long-range molecular order.^36,37^Fig. 3d displays the *quasi in situ* Raman evolution of spectra of freeze-dried samples at different times of synthesis. A spectrum of the fully crystallized silicalite-1 sheet stacks upon calcination is added as reference (ESI Fig. 10†).

**Fig. 4:**
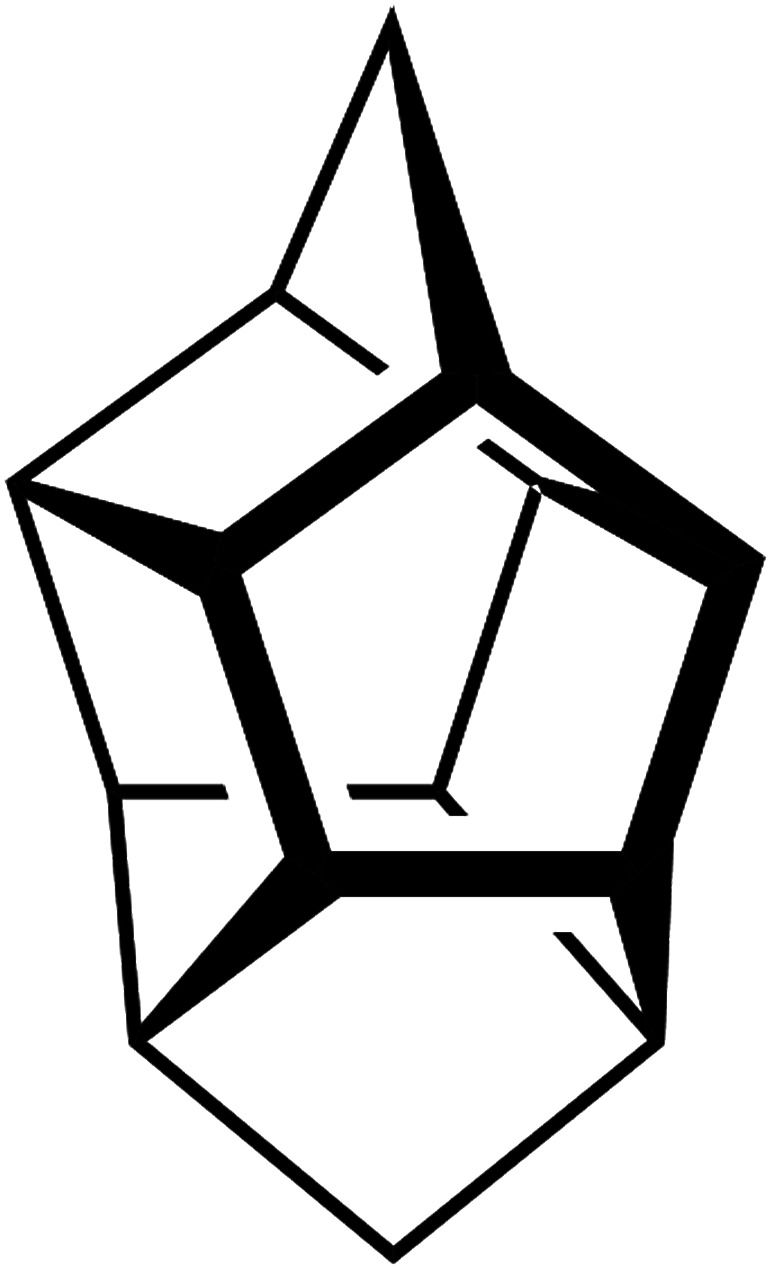
The pentasil unit as found in silicalite-1 zeolite with the MFI topology.

Whereas the majority of bands come from the DQAS SDA, early presence of zeolitic features in the solids is unambiguous, as witnessed by bands at 516 cm^−1^ and 380 cm^−1^.^38^ The former band corresponds to the vibration of 4-membered rings, and is prominently present at the very early times of synthesis. The latter band belongs to larger, 5-membered silicate rings that characteristically structure silicalite-1 (Fig. 4). This early presence of silicate double-5-rings is also witnessed by comparable *quasi in situ* infrared spectroscopy experiments that reveal a band at 550 cm^−1^ (ESI Fig. 2†).

In Raman, the stretching of this unit is also visible at very early synthesis times (yet, weakly), and intensifies over the course of hydrothermal synthesis, where as we know, molecular organisation towards bulk crystallinity makes headway.

All in all, it is clear that structural features of silicalite-1 are present at very early synthesis time, and even at time-zero, which indicates that the DQAS SDA is highly effective in stabilizing zeolitic precursor species.

**Fig. 5:**
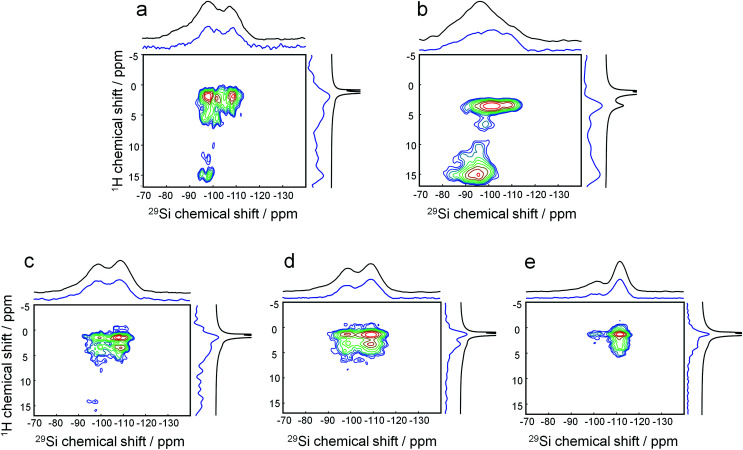
Evolution of template-silica interactions. ^29^Si {^1^H} HETCOR NMR spectra of freeze-dried samples: (a) room temperature aged C_22-6-3_-silica gel, (b) aged CTAB-silica gel and hydrothermally treated silicalite-1 nanosheets for (c) 12 h, (d) 24 h, and (e) 72 h. The independently measured one dimensional ^1^H and ^29^Si {^1^H} CPMAS spectra are plotted on top of the projections.

*Quasi in situ* solid-state NMR can reveal how this molecular structure direction is established:

^1^H–^29^Si HETCOR MAS NMR on freeze-dried samples (analogous to the aforementioned experiments) shows that after mixing at room temperature, the headgroup of the DQAS C_22-6-3_ already resides within the silica matrix (Fig. 5a). This is witnessed by the fact that methylene protons in β-position with respect to diquaternary ammonium render cross-peaks.^39^ Comparing this to a similar mixture with CTAB instead of the DQAS is interesting, because we know that the former fails at directing molecular structure, and non-crystalline MCM-41 materializes. Indeed, the β-positioned methylene protons do not produce cross-peaks in the case of CTAB (Fig. 5b). Whereas the CTAB headgroup is at the silica–water interface (evidenced by the methyl proton-silica cross-peak), it is not *within* the silica matrix.

The broad cross-peaks at very low fields (*δ*(^1^H) > 10 ppm) are due to SiO–H–OSi bridges, typically associated with disordered condensation, and at longer synthesis times, defects. It is notable that these cross-peaks come much more diffuse and at higher intensity for the synthesis with CTAB as template, underlining its inability to direct molecular structure in the condensed phase under these conditions. The two resonances in the ^29^Si dimension correspond to (tetrahedral) silica to which attached is one terminal OH ligand and three bridging O ligands, denoted Q_3_ around −95 to −100 ppm, and silica to which only bridging O is attached, Q_4_ at −105 to −110 ppm.

As we heated the DQAS-templated mixture and proceeded *quasi in situ*, the evolution of ^1^H–^29^Si HETCOR MAS NMR spectra with synthesis time (Fig. 5c–e) shows that the initial defects disappear during nanosheet formation, concomitant with a decrease of the Q_3_ : Q_4_ ratio in proceeding silica condensation.

Deconvolution of direct-excitation ^29^Si MAS NMR spectra (ESI Fig. 3†) reveals a Q_2_^29^Si resonance, and allows for quantification of the Q_2_ : Q_3_ : Q_4_ ratio by peak integration. Over the course of 72 h hydrothermal synthesis, Q_2_ : Q_3_ : Q_4_ ratios change from 26 : 34 : 40, from time-zero, to 9 : 29 : 62 after 72 h of hydrothermal synthesis (ESI Table 2†). Calcination, as was also seen by Raman scattering, further enhances long-range molecular order, herein corroborated by further sharpening of the ^29^Si resonances, as well as further relative intensifying of the Q_4_ signal (Q_2_ : Q_3_ : Q_4_ = 7 : 9 : 84). Overall, this indicates that the formation of a ‘hard’ framework requires prolonged heating and calcination. This was also witnessed in TEM in which the sample aged at room temperature swiftly broke down into spherical silica particles upon exposure to the electron beam (ESI Fig. 4†), whilst sheets obtained after hydrothermal treatment were much more stable during the EM imaging (TEM frame-based were uploaded as ESI†).

In order to further verify that molecular recognition between DQAS SDA and silica takes already place at the early stages of synthesis, we synthesized a modified version of the C_22-6-3_ DQAS in which the methyl side-groups are replaced by propyl side-groups: this SDA will be referred to as C_22-6(3)-3(3)_. The use of this DQAS in an otherwise unchanged synthesis gel resulted in formation of thin, needle-like silicalite-2 crystals (this followed from XRD and SEM, Fig. 7 and ESI Fig. 5†). Silicalite-2 is of MEL topology, which is only subtly different from MFI topology, and is in comparison built up from Si_33_ building units that contain 4- and 6-membered rings along the large 10-member end rings (the Si_33_ units found in silicalite-1 only contain 5- and 10-membered rings).

**Fig. 6:**
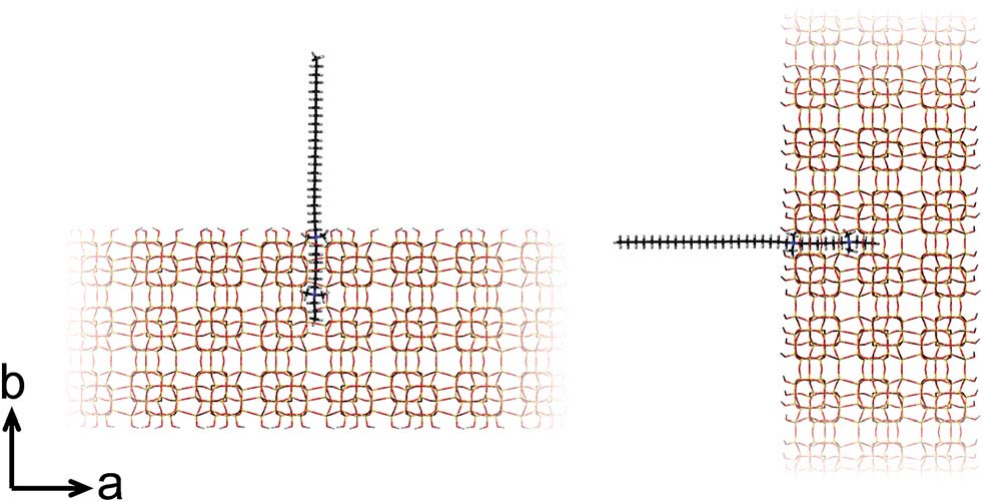
Graphic representation of the alignment of C_22-6-3_ in the (010) (left) and (100) (right) directions of silicalite-1.

At this point, our analysis begs for further investigation by computation. We proceed with force-field based static and molecular dynamics simulations to investigate DQAS SDA interaction with silicalite-1 and silicalite-2. Let us first move to bulk nanosheet models. Herein we took into account the effect of the DQAS headgroup environment by studying C_22-6-3_ and C_22-6(3)-3(3)_. Now, taking the silicalite-1 and silicalite-2 lattices into account, we investigated the interaction energy with the DQAS SDAs inserted into both (010) and (100) planes of silicalite-1 (Table 1 and Fig. 6). Both insertions are, in principle, sterically viable, yet the former configuration is the experimental result. The configurations with the SDAs in silicalite-2 lattices in the (100) and (010), also given in the table, are equivalent.

**Table 1:** Interaction energies of different SDA with MFI and MEL surfaces. Values in kJ mol^−1^

Structure (surface)	C_22-6-3_	C_22-6(3)-3(3)_
Silicalite-1 (010)	−569	−519
Silicalite-1 (100)	−586	−661
Silicalite-2 (100/010)^a^	−548	−531

a The (100) and (010) directions are equivalent within the *I*4̄*m*2 space-group.

As can be seen from Table 1, template–silica framework interaction energies are very comparable for both the (010) and (100) configurations, with in fact the latter a tad more stabilizing. We derive from this that specific orientation of C_22-6-3_ is kinetically regulated and adopted at earlier stages of the synthesis (which is in line with our spectroscopic analysis).

To understand how silicate is structured in pre-organised zeolitic entities, we continued our molecular study on the Si_33_ building units of the silicalite-1 and silicalite-2 structures. The role of Si_33_ as precursor entity has been speculated on without rock-solid evidence as yet, but earlier modelling did show that these units are stabilized by tetrapropylammonium (TPA^+^).^27^

In these studies on silicalite-1 zeolite formation, it was demonstrated how the MFI–TPA^+^ composite can be assembled from Si_33_ units in the presence of TPA^+^ as a structure-directing agent. In addition, considering the confirmed existence of 5-membered species at time-zero (see above), a computational approach with the Si_33_ unit as putative building block appears a reasonable model to study silica organization at early synthesis times.^40^Table 2 lists average interaction energies between C_22-6-3_/C_22-6(3)-3(3)_ with the Si_33_ units. Here we see that Si_33_, as extracted from equilibrated molecular dynamics simulations, prefers to reside perpendicularly to the SDA axis, in between both quaternary groups. If Si_33_ is placed along the template axis at either side of both the quaternary ammonium groups, the SDA–silica interaction becomes substantially less stabilizing.

**Table 2:** Computer simulations showing the interaction between the DQAS and Si_33_ building units.^a^ Values in kJ mol^−1^

	Si_33_-MFI, C_22-6-3_	Si_33_-MFI, C_22-6(3)-3(3)_	Si_33_-MEL, C_22-6-3_	Si_33_-MEL, C_22-6(3)-3(3)_
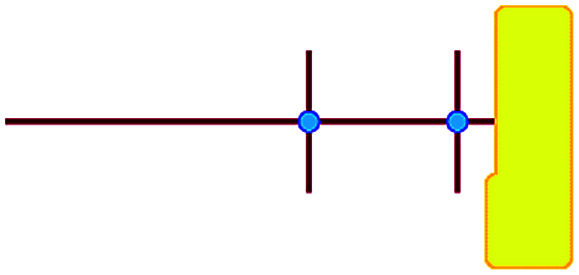	−186	−126	−155	−155
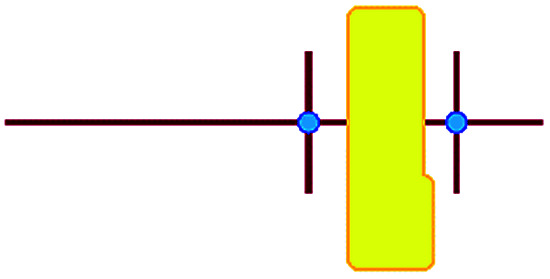	−478	−521	−476	−519
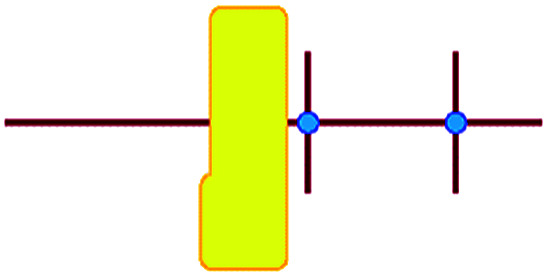	−213	−215	−178	−191
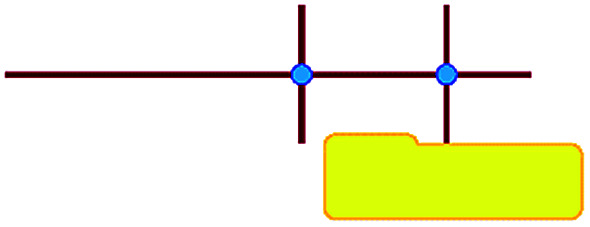	n/a^b^	−247	n/a^a^	−246

a
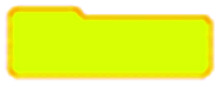
 represents putative Si_33_ units for MFI and MEL zeolite; the red lines the alkyl chains and the blue dots the quaternary ammonium centers in the DQAS; energies of stable configurations in kJ mol^−1^ are given for the interaction of silicalite-1 (MFI) and silicalite-2 (MEL) Si_33_ building units with DQAS C_22-6-3_ and C_22-6(3)-3(3)_.

b Not available (no stable configuration identified).

**Fig. 7:**
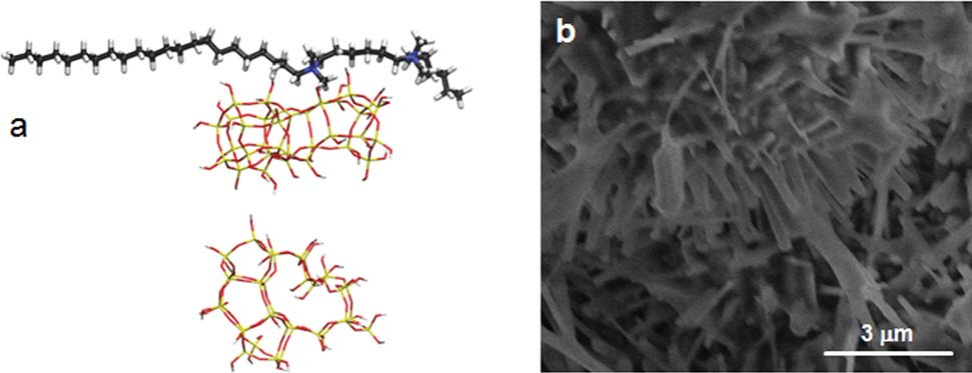
(a, top) The Si_33_ unit of MFI placed laterally towards the SDA and (a, bottom) the structure of Si_33_ with the SDA removed for clarity, showing the collapse of Si_33_ during the MD simulations. (b) SEM image of MEL needles obtained in the C_22-6(3)-3(3)_-silica system.

If initially placed close, and lateral, to one of the methyl side groups of C_22-6-3_, Si_33_ loses interaction with the template, and the ring structure collapses (Fig. 7a). The reason for this is that the (quaternary ammonium-bound) methyl group is too short to stabilize the Si_33_ structure – it has been shown before that the alkyl chain must be sufficiently long to stabilize the hydrophobic Si_33_ unit during MFI formation.^40^

Thus, early stabilization of C_22-6-3_-Si_33_ units leads to assembly of a (010) lattice, in which Si_33_ units are embedded in an extended lattice of nanosheets with a very short *b*-axis. We now also understand the formation of silicalite-2: the use of C_22-6(3)-3(3)_ inhibits growth in (100) and (010) directions. These directions are equivalent within the *I*4̄*m*2 space-group, and there is no insertion of the SDA DQAS possible in the (001) direction. The result is thus that silicalite-2 forms in the form of needle-like crystals (Fig. 7b). This projects an exciting possibility towards the *crystal engineering* of stacked-sheet, silicate materials by subtly changing the headgroup environment of DQAS SDAs.

## Conclusions

In applying a multi-scale spectroscopic and computational approach, we demonstrated that the synthesis of stacked silicalite-1 nanosheets proceeds through a pre-organised phase in the solid-state. Most remarkably, this phase adopts its meso-scale (stacking) order of the final material already within the first five hours of synthesis.

At the molecular level, the phase already contains *zeolitic* structural features. This is the consequence of molecular recognition of specific silicate species by the anisotropically distributed hydrophobic functionalities of the DQAS template. We further demonstrated how molecular recognition can be tuned in order to direct topology and aspect ratios of the material's crystals.

This work provides some necessary rationale towards hierarchical zeolite synthesis. We have shown that meso-scale order is established well before long-range molecular order occurs. Nevertheless, molecular recognition at early synthesis times, stabilizing zeolitic precursor units appears a requisite, and in order to establish this, the DQAS SDA must reside within the silica matrix from the earliest of synthesis times.

We expect that the insight from this work will help the development of tailored and inexpensive SDAs to direct synthesis of (new) hierarchically structured zeolite materials.

## Synthetic procedures, materials and methods

### Sample preparation

The synthesis of silicalite-1 nanosheets starts with the dissolution of the bromide form of the diquaternary ammonium surfactant (DQAS), C_22_H_45_–N^+^(CH_3_)_2_–C_6_H_12_–N^+^(CH_3_)_2_–C_3_H_7_ (C_22-6-3_), and NaOH (EMSURE, 50 wt%) in water, followed by stirring at 60 °C for 1 h to obtain a clear solution. We have recently shown that replacing the hexyl end group of the original DQAS surfactant used by Ryoo and co-workers^15^ by a propyl end group increases the rate of zeolite nanosheet crystallization.^20^ After cooling to room temperature, TEOS (tetraethyl orthosilicate, Merck, 99%) was quickly added. The resulting suspension with a gel composition of 9C_22-6-3_ : 100SiO_2_ : 11Na_2_O : 4000H_2_O was stirred for 1 h at 40 °C. The reference zeolite was synthesized by placing this suspension in a Teflon-lined autoclave and heating the closed autoclave to 150 °C for 7 days. In further synthesis experiments, similar suspensions were placed in a similar autoclave at 135 °C rotated at 50 rpm for varying times to obtain solids for further characterization. These solids were obtained by freeze-drying for 24 h. Template was removed by calcination in air with a heating ramp of 1 °C min^−1^ to 550 °C and kept at that temperature for 8 h.

### Sample characterization

The solids were characterized by XRD, electron microscopy, NMR, and Raman and infrared spectroscopy. Aliquots of the synthesis gels after autoclaving at 135 °C for varying times were freeze-dried for 24 h and investigated by transmission and scanning electron microscopy. Small-Angle X-ray Scattering (SAXS) was employed to follow the development of structures at the mesoscale. An *in situ* cell specifically designed for this purpose^32^ was used to record SAXS patterns at the Dutch-Belgian Beamline (DUBBLE) of the ESRF synchrotron in Grenoble. The patterns were recorded at room temperature and at 135 °C under rotation.

The synthesis of the SDAs and detailed information about the characterization methods is described in the ESI.†

## Supplementary Material

SC-007-C6SC01295G-s001

SC-007-C6SC01295G-s002

SC-007-C6SC01295G-s003
