# Supplementary material for: Establishing hierarchy: the chain of events leading to the formation of silicalite-1 nanosheets
Source: Chem Sci. 2016 Jun 22;7(10):6506–13. doi: 10.1039/c6sc01295g (PMC5458680; doi:10.1039/c6sc01295g)
Supplement: SC-007-C6SC01295G-s001 [file SC-007-C6SC01295G-s001.pdf]

Electronic Supplementary Material (ESI) for Chemical Science.
This journal is © The Royal Society of Chemistry 2016

SUPPLEMENTARY INFORMATION

Establishing hierarchy: the chain of events leading to the formation of Silicalite-1 nanosheets

Xiaochun Zhu, Maarten G. Goesten, Arjan J.J. Koekkoek, Brahim Mezari, Nikolay Kosinov, Georgy Filonenko, Heiner Friedrich, Roderigh Rohling, Bartłomiej M. Szyja, Jorge Gascon, Freek Kapteijn and Emiel J.M. Hensen

	PAGE
SUPPLEMENTARY METHODS	2
SUPPLENTARY FIGURES	7
SUPPLEMENTARY TABLES	14
SUPPLEMENTARY REFERENCES	17

SUPPLEMENTARY METHODS

1. Synthesis of surfactants

Synthesis of $C_{22}H_{45}$-$N^{+}(CH_3)_2$-C_6H_{12}-$N^{+}(CH_3)_2$-C_3H_7 (C_{22-6-3})

3.9 g (0.01 mol) of 1-bromodocosane (TCI, 98 %) was dissolved in 50 ml toluene (Biosolve, 99.5 %) and added dropwise into a mixture of 50 ml acetonitrile (Biosolve, 99.8 %) and 21.4 ml (0.1 mol) N,N,N',N'-Tetramethyl-1,6-hexanediamine. The reaction was refluxed in an oil bath at 70 °C for 12 h. The solution was cooled to room temperature, which led to the precipitation of a white solid. The suspension was further cooled at 5°C for 1 h. The white solid (N-(6-(dimethylamino)hexyl)-N,N-dimethyldocosan-1-aminium bromide; C_{22-6}·Br) was filtered and washed with diethyl ether (Biosolve, 99.5 %).

Then, 5.6 g of C_{22-6}·Br and 2.5 g of 1-bromopropane (Aldrich, 99 %) was dissolved in 100 ml acetonitrile and 10 ml ethanol. The solution was refluxed in an oil bath at 70 °C for 12 h. After cooling to room temperature, the white solid (C_{22-6-3}·Br_2) was filtered and washed with diethyl ether. The product was dried overnight at 50 °C under vacuum.

Synthesis of $C_{22}H_{45}$-$N^{+}(CH_3)_2$-C_6H_{12}-$N^{+}(CH_3)_2$-C_3H_7 ($C_{22-6(3)-3(3)}$)

30 g of 1,6-dibromohexane was mixed with 200 ml of dipropyl amine. The solution was refluxed at 75 °C for 48 h. After cooling, during which a white solid precipitated, the solution was neutralized with 0.5 l diluted sodium hydroxide solution (0.5 M). The organic products were thrice extracted with 150 ml diethyl ether and the combined organic extracts were dried over $MgSO_4$. The volatile organics were removed on a rotary evaporator, yielding a slightly yellow oil (N,N,N',N'-Tetrapropyl-1,6-hexanediamine).

12 g of N,N,N',N'-Tetrapropyl-1,6-hexanediamine and 1.8 g of bromodocosane were dissolved in 100 ml acetonitrile/toluene (1:1) and the solution was refluxed for 12 h at 70 °C. The volatile compounds of the synthesis mixture were removed on a rotary evaporator under reduced pressure. The remaining products were dispersed in diethyl ether and the solid white product was recovered by filtration and washed with copious amounts of diethyl ether yielding N-(6-(dipropylamino)hexyl)-N,N-dipropyldocosan-1-aminium bromide ($C_{22-6(3)}$·Br) as a white solid product. Then, 10 g of $C_{22-6(3)}$·Br and 3.6 g of 1-bromopropane (Aldrich, 99 %) were dissolved in 100 ml acetonitrile and 10 ml ethanol. The solution was refluxed in an oil

bath at 70 °C for 12 h. After cooling to room temperature, the white solid ($C_{22-6(3)-3(3)}\cdot Br_2$) was filtered and washed with copious amounts of diethyl ether. The product was dried overnight at 50 °C under vacuum.

2. Sample preparation

CTAB-silica precursor. For comparison, an MCM-41 precursor was prepared according to the konwn procedure. Cetyltrimethylammonium bromide (CTAB, Aldrich 95 %) and NaOH (EMSURE, 50 wt%) were dissolved in water, followed by stirring at 60 °C for 1 h to obtain a clear solution. After cooling to room temperature, TEOS (Merck, 99 %) was quickly added. The resulting suspension was stirred for 1 h at 40 °C. The final gel with a gel composition of 9 CTAB : 100 SiO_2 : 11 Na_2O : 4000 H_2O was freeze-dried for 24 h.

MEL Zeolite. The $C_{22-6(3)-3(3)}$ template (bromide form) NaOH (EMSURE, 50 wt%) was dissolved in water at 60 °C. The template solution was cooled to RT. Then TEOS (Merck, 99 %) was quickly added. The suspension was vigorously stirred for 1 h in an open vessel at room temperature and subsequently transferred to a Teflon-lined stainless steel autoclave. The autoclave was heated under rotation at 150 °C for 7 days. The white product was recovered by filtration and washed with copious amounts of water and ethanol. The product was dried at 110 °C.

3. Characterization

Small-angle X-ray scattering (SAXS). *In-situ* SAXS analysis was carried out at the DUBBLE beamline at the European Synchrotron Radiation Facility (ESRF) in Grenoble (beamline BM26B). The solutions described above are loaded into a homemade cell, which is heated up to 135°C under rotation to prevent sedimentation. Small-angle (SAXS) and wide-angle (WAXS) patterns were acquired simultaneously. Two Pilatus photon counting detectors are used to collect the 2D-images. SAXS images were collected using a Pilatus 1M (169 mm x 179 mm active area). WAXS patterns were collected using a 300K linear Pilatus detector (254 mm x 33.5 mm active area), to monitor the eventual appearance of 'real' crystallinity, which was in no case found. In both cases, spectra were taken at scan times of 100 seconds, with 200 seconds 'rest', effectively taking a spectrum every 5 minutes.

The fittings in figure 2A of the manuscript were performed with a form factor *P(q)* for a (planar) sheet:

$$P(q,\eta,L) = \left[\eta \cdot L \cdot \frac{\sin(\frac{qL}{2})}{qL/2}\right]^2$$

with η the sheet thickness and L the lateral dimension. Sheet stacking was fitted with the Paracrystalline Structure factor $S(q)$:

$$\lim_{\delta \to d} S_{PT} = N_k + 2 \sum_{m=1}^{N_k - 1} (N_k - m)\, cos(mqd)\, exp(-\frac{m^2 q^2 \delta^2}{2})$$

with N_k the number of sheets, d the distance between the sheets in nm, and δ the stacking disorder in nm, a root-mean square deviation of the stacking distance.

We now demonstrate that ω, the order parameter in the main text, acts as a true factor for realistic systems, as for $\delta \to d$, the mathematical term that governs the structure factor

$$cos(mqd) exp(-\frac{m^2 q^2 d^2}{2})$$

converges rapidly to zero for

$$q > {}^{2\pi}/_{md}$$

where m is the number of repeating units, and d the d-spacing. This condition implies that for realistic paracrystalline materials, the structure term vanishes already at q-values of the “fractal regime”, where quasi-Bragg peaks do not occur. For example, for m = 4 and d = 5 nm, S_{PT} vanishes for q > 0.31.

XRD. X-ray diffraction patterns were recorded on a Bruker D2 PHASER using Cu Kα radiation in the 2θ range of 5−60 ° with a step size of 0.02 ° and a time per step of 0.4 s.

Electron microscopy. Scanning electron microscopy (SEM) images were taken on a FEI Quanta 200F scanning electron microscope at an accelerating voltage of 3 kV using the secondary electron detector. The zeolite samples which were deposited onto SEM stubs and were sputter-coated with gold (thickness about 2 nm) prior to the measurements. Transmission electron microscopy of dry samples was performed

on a Tecnai 20 (FEI company). The microscope contains a LaB_6 electron gun and a TWIN objective lens operated at an acceleration voltage of 200 kV. Images were recorded on a Gatan model 794 1k×1k ‘slow scan’ CCD camera. TEM samples were prepared from suspension in ethanol of which a few drops were placed onto a standard 200 mesh copper TEM grid coated with a holey Carbon film. The samples were dried at room temperature prior to insertion in the TEM holder and microscope.

Vibrational spectroscopy. UV Raman spectra were recorded with a Jobin-Yvon T64000 triple stage spectrometer with spectral resolution of 2 cm^{-1} operating in double subtractive mode. The laser line at 325 nm of a Kimmon He-Cd laser was used as to excite the sample. The power of the laser at the sample position was 4 mW. FT-IR spectra of samples were recorded on a Bruker Vertex 70v instrument. The spectra were acquired at 2 cm^{-1} resolution and 64 scans. IR Spectra were normalized by the weight of the catalyst wafer.

NMR spectroscopy. One dimensional ^{1}H, $^{29}Si\{^{1}H\}$ cross polarization (CP) and two-dimensional $^{29}Si\{^{1}H\}$ heteronuclear correlation (HETCOR) Nuclear Magnetic Resonance (NMR) spectra were recorded on an 11.7 Tesla Bruker DMX500 NMR spectrometer, operating at 500 MHz for ^{1}H and 99 MHz for ^{29}Si measurements. Quantitative Direct-Excitation (DE) ^{29}Si NMR spectra were recorded on an 4.7 Tesla Bruker DRX200 NMR spectrometer, operating at 200 MHz for ^{1}H and 39.7 MHz for ^{29}Si measurements. The measurements were carried out using 4 mm magic-angle-spinning (MAS) probe heads, on both magnets, with sample rotation rate of 10 kHz. ^{1}H NMR spectra were recorded with a Hahn-echo pulse sequence p_1-τ_1-p_2-τ_2-aq with a 90° pulse p_1 = 5 μs and a 180° p_2 = 10 μs, and an interscan delay of 5 s. 1D $^{29}Si\{^{1}H\}$ CPMAS and 2D $^{29}Si\{^{1}H\}$ HETCOR NMR spectra were recorded with a rectangular contact pulse of 3 ms, with carefully matched amplitudes on both channels, and an interscan delay of 3 s. ^{1}H NMR ^{29}Si shifts were both calibrated using tetramethylsilane (TMS). For DE ^{29}Si NMR spectra, 128 aquisitions were accumulated with an interscan delay of 720 seconds. The spectra were deconvoluted using Gaussian line shapes in DMfit2011 program.[1]

4. Molecular modelling

Biovia Material Studio 6.0 was used for theoretical modeling. The interaction energy of the DQAS with MFI and MEL was determined by embedding the DQAS into the surfaces with the long hydrocarbon tail pointing outwards and one quaternary ammonium ion fully embedded in the zeolite framework and the other at the surface. Periodic boundary conditions (PBC) were applied. The COMPASS force field was used to optimize the geometry.

To study the interaction energies of Si_{33} building units with the DQAS and the stability of the precursor-DQAS complexes in solution, large cubic boxes of 50 Å dimension (PBC) with explicit modelling of 4000 water molecules were constructed. For all molecular dynamics runs, the Forceite+ module was used in combination with the COMPASS force field. The models were optimized using the NPT ensemble simulating 65 ps at 1 bar and 298 K using the Berendsen and Nosé baro- and thermostats, respectively. A typical production run was performed using the NVT ensemble for 600 ps at 298 K using the Nosé thermostat. All runs were performed with 1 fs time steps and a 12.5 Å cut-off distance for both the Van der Waals non-bonded and the electrostatic interactions using atom-based summation methods. The trajectories were analysed with the help of an in-house written PERL script. The script was used to calculate the potential and interaction energies of the system after removal of the water molecules, because the presence of water increased the standard deviation of the energy values significantly. The charge of the SDA cations was balanced by deprotonating the Si_{33}-precursors.

SUPPLEMENTARY FIGURES

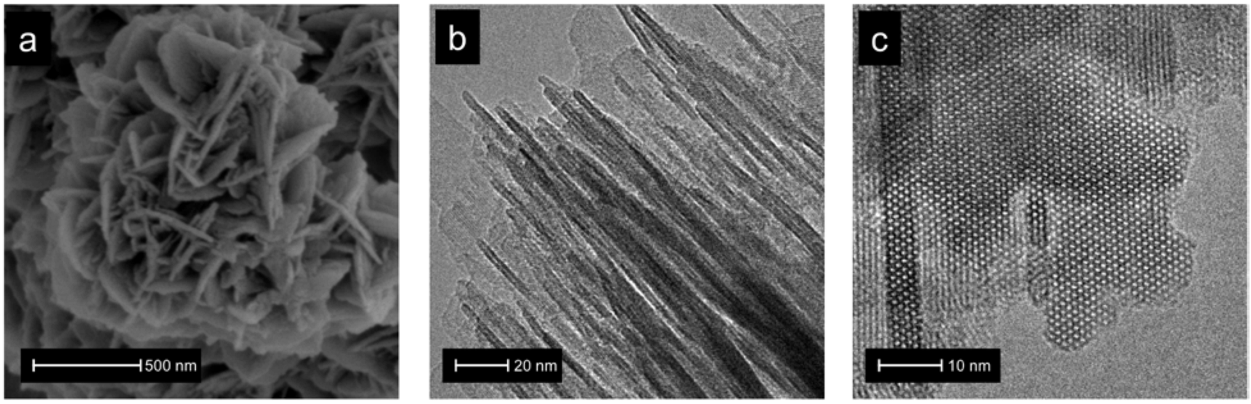

Supplementary Figure 1 | (a) SEM image and (b, c) TEM images of fully crystalline silicalite-1 nanosheets obtained after hydrothermal synthesis at 150 °C for 7 days.

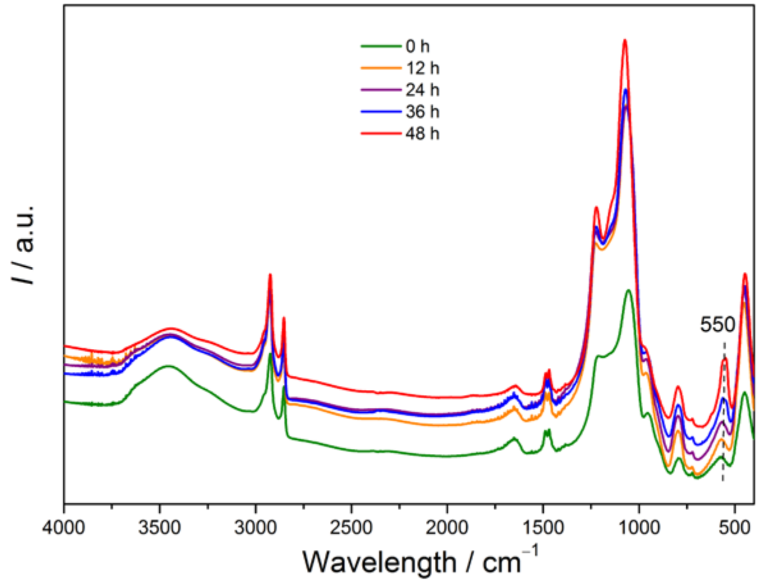

Supplementary Figure 2 | FTIR spectra of silicalite-1 nanosheets as function of synthesis. The band at 550 cm^{-1} is related to the presence of D5R rings, characteristic for MFI zeolite.[2]

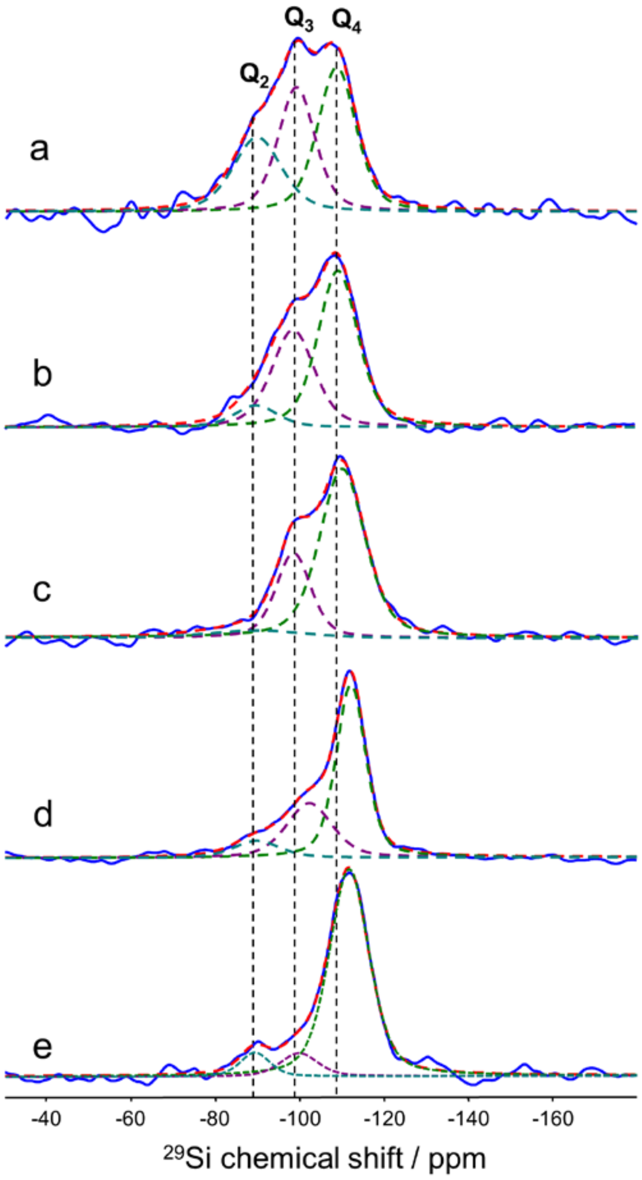

Supplementary Figure 3 | Direct-excitation ^{29}Si MAS NMR spectra of the freeze-dried samples: (a) aged $C_{22\text{-}6\text{-}3}$-silica gel, and after hydrothermal synthesis for (b) 12 h, (c) 24 h, and (d) 72 h and (e) 72 h sample after template removal by calcination.

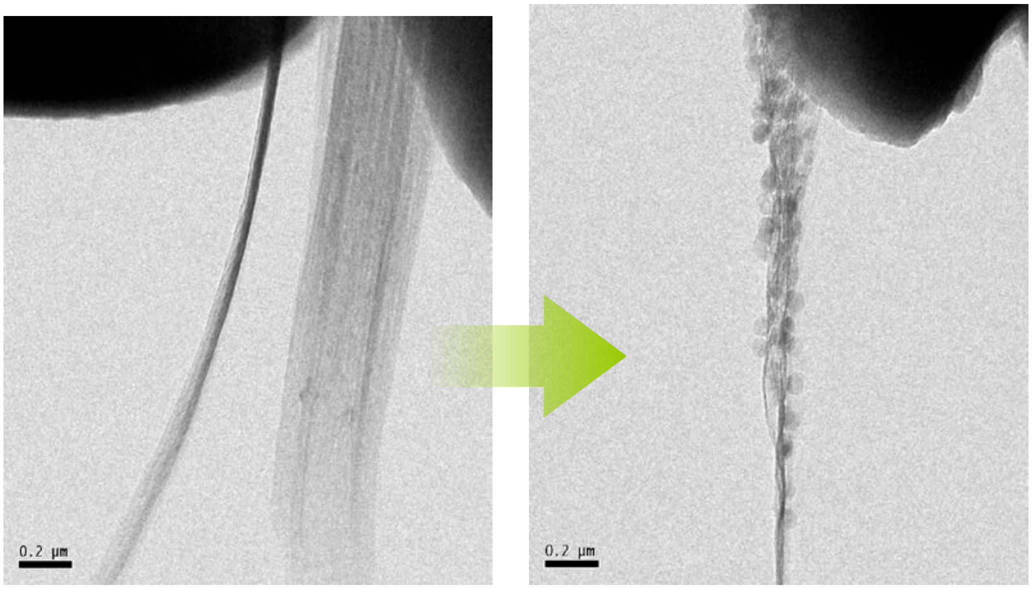

Supplementary Figure 4 | Transmission electron microscopy images of room-temperature aged $C_{22\text{-}6\text{-}3}$-silica gel (left) directly after exposure to the electron beam and (right) after 2 min, displaying the softness of the early sheets.

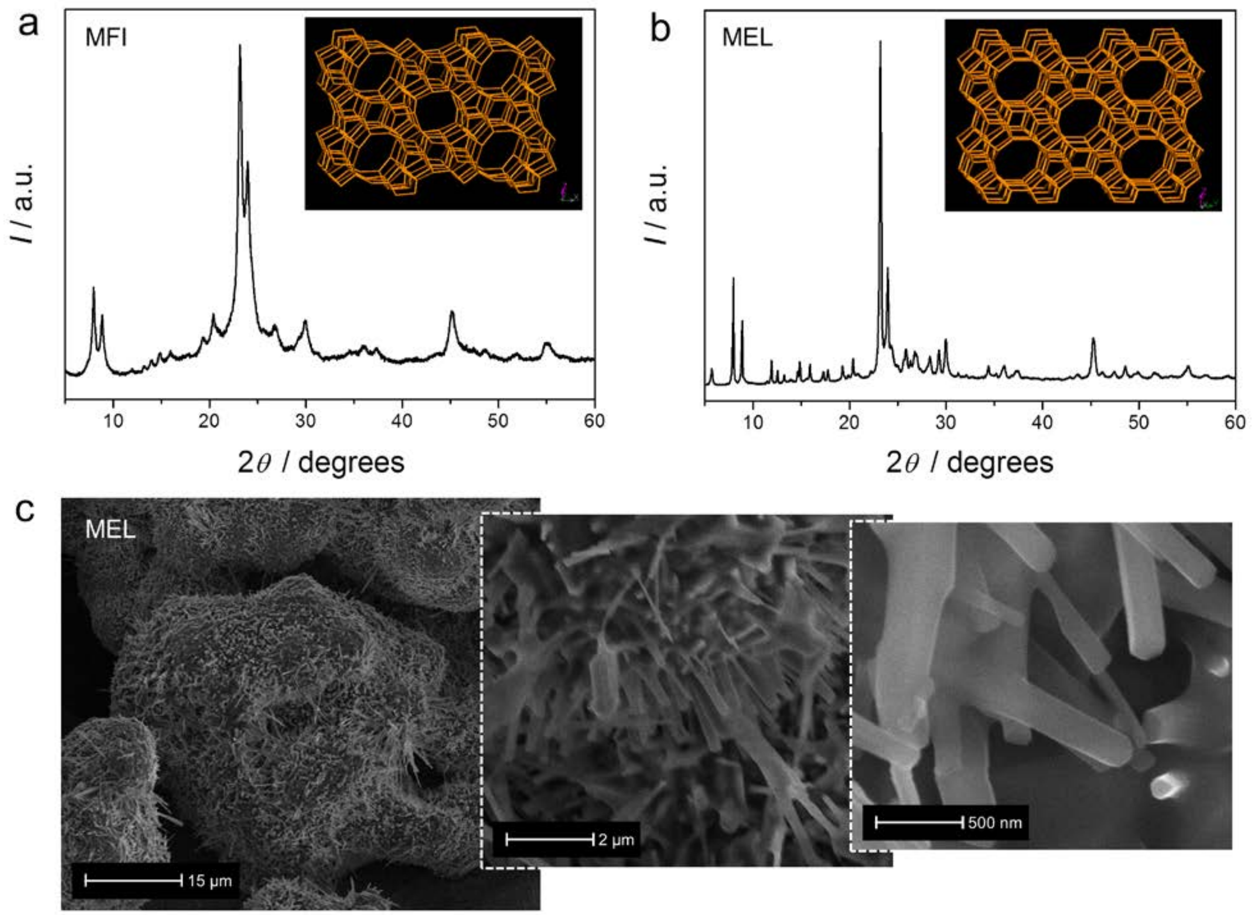

Supplementary Figure 5 | XRD patterns of silicalite-1 nanosheets (a) and MEL zeolite (b). (c) SEM images of MEL zeolite synthesized with DQAS surfactant $C_{22\text{-}6(3)\text{-}3(3)}$.

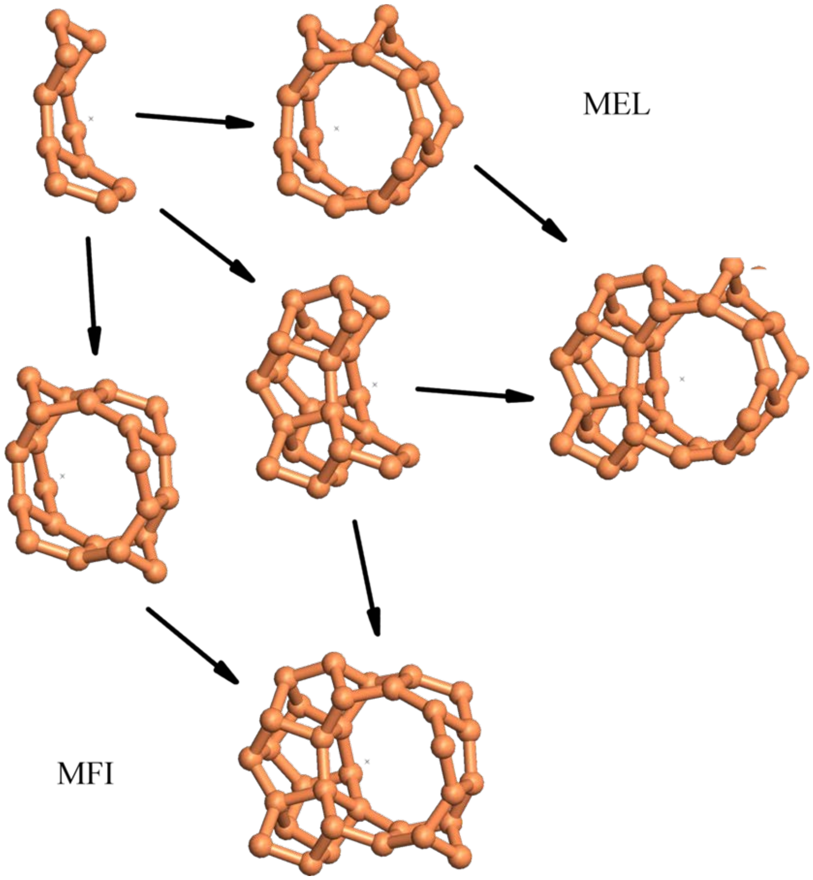

Supplementary Figure 6 | Assembly of Si_{11} units into Si_{22} and Si_{33} building units that are comprised in the framework of MFI and MEL zeolites. The Si_{11} and Si_{22} units that are common to MEL and MFI contain only 5-membered rings in their structure. One 4-membered and one 6-membered ring are formed upon symmetric (mirror plane) agglomeration of two Si_{11} units to form the initial channel – or 10 ring – in the MEL Si_{22} unit. The 4- and 6-membered rings of MFI can only be formed in the subsequent step of the process, where the Si_{33} units agglomerate.

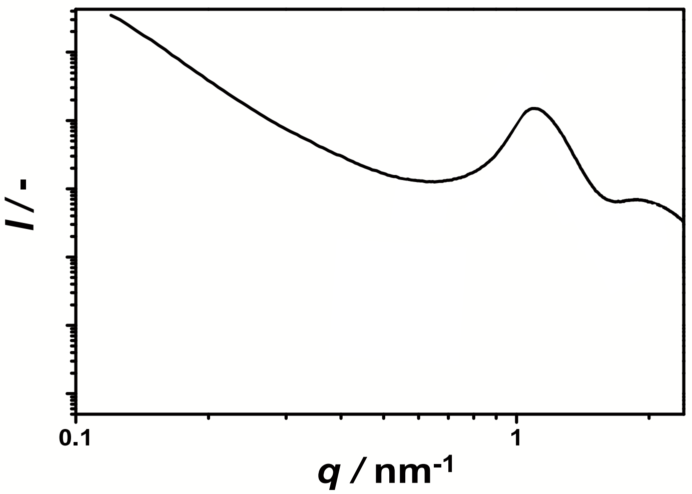

Supplementary Figure 7 | A SAXS pattern obtained after ageing a C_{22-6-3} templated system at room temperature, before heating. The *quasi*-Bragg peak, described in the main text, is already present.

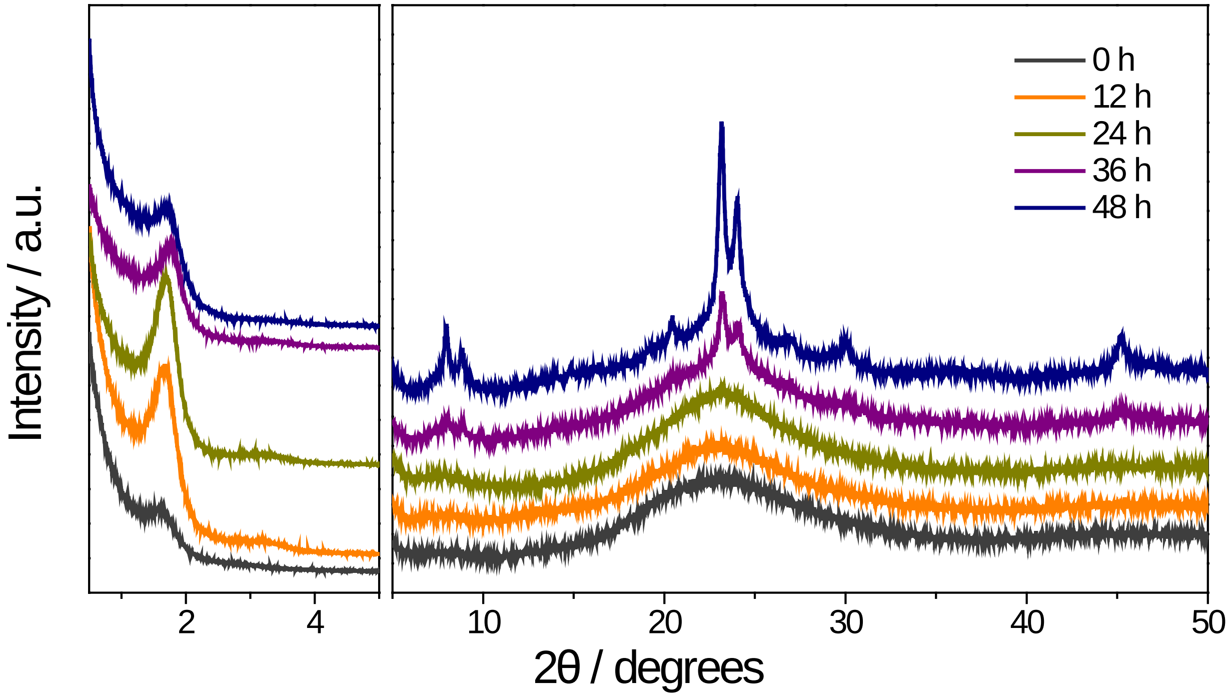

Supplementary Figure 8 | Quasi *in-situ*, XRD patterns of the crystallization of Silicalite-1, as templated by $C_{22\text{-}6\text{-}3}$. The low-angle region, which shows the basal reflections, is highlighted on the left.

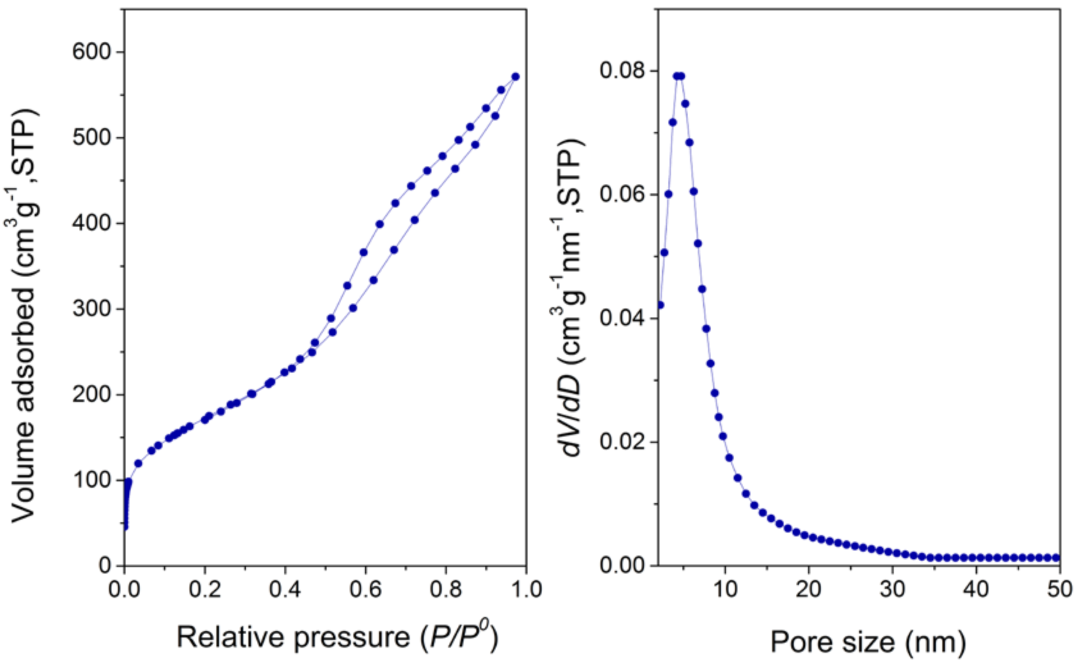

Supplementary Figure 9 | Ar sorption isotherm of calcined Silicalite-1 nanosheets, obtained with $C_{22\text{-}6\text{-}3}$ (BET surface area: 550 m^2 g^{-1}; total pore volume 0.73 cm^3 g^{-1}; NLDFT micropore volume 0.12 cm^3 g^{-1}; BJH mesopore volume 0.63 cm^3 g^{-1}).

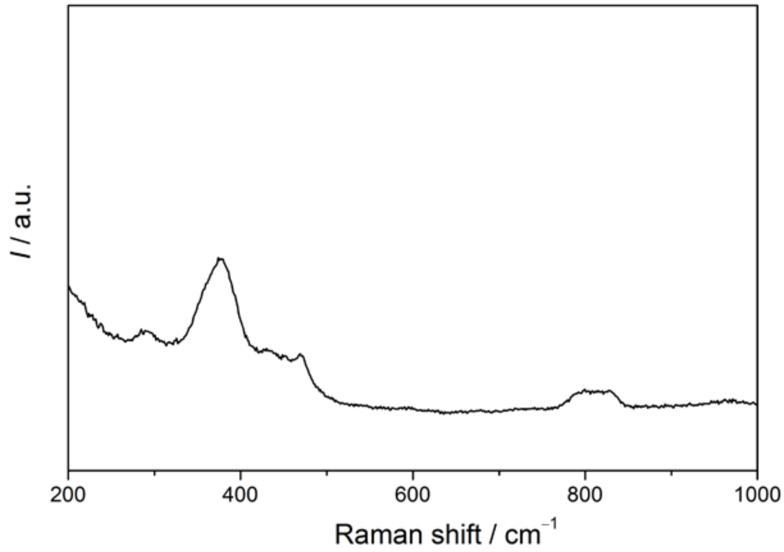

Supplementary Figure 10 | The Raman spectrum of fully crystallized, calcined Silicalite-1 nanosheets, with bands at 290 cm^{-1} (10-membered rings), 380 cm^{-1}, 470 cm^{-1}, and 800 cm^{-1}.[3,4]

SUPPLEMENTARY TABLES

Supplementary Table 1 | Fitting values for the SAXS data; D_{core} is the thickness of the sheet, N the number of sheets, d the inter-sheet distance, the stacking disorder, diffuse (additional) scattering and the calculated goodness of fit (GOF).

Time (min)	D_{core} (nm)	N	d (nm)	δ (nm)	v	*GOF* (%)
263	1.5	14.2	4.43	0.42	0	99.51
243	1.5	13.5	4.42	0.42	0	99.52
223	1.48	12.5	4.41	0.43	0	99.56
203	1.49	11.9	4.41	0.43	0	98.71
183	1.49	11.5	4.40	0.44	0	99.57
163	1.48	9.7	4.39	0.44	0	98.60
143	1.48	10.2	4.39	0.44	0	99.57
123	1.48	9.4	4.39	0.45	0	99.61
103	1.48	9.0	4.38	0.45	0	99.58
83	1.48	8.4	4.37	0.47	0	99.64
63	1.46	8.3	4.37	0.47	0	99.63
43	1.46	6.9	4.37	0.48	0	99.60
23	1.46	6.0	4.37	0.48	0	99.57
3	0.7	5.0	4.37	0.47	0	99.74

Supplementary Table 2 | Deconvolution of direct-excitation ^{29}Si MAS NMR spectra shown in Supplementary Fig. 4.

Sample	Q_2 (%)	Q_3 (%)	Q_4 (%)
aged $C_{22\text{-}6\text{-}3}$-silica gel	26	34	40
135 ºC; 12 h	8	37	55
135 ºC; 24 h	6	26	68
135 ºC; 72 h	9	29	62
135 ºC; 72 h; calcined	7	9	84

Supplementary Table 3 | Stabilization of different $(SiO)_n$ rings by TPA with TPA inside and outside the ring structure (energies in kJ/mol)

	TPA (inside)	TPA (outside)
4-ring	-771	-349
5-ring	-670	-378
6-ring	-763	-360

SUPPLEMENTARY REFERENCES

1. D. Massiot, F. Fayon, M. Capron, I. King, S. Le Calvé, B. Alonso, J.-O. Durand, B. Bujoli, Z. Gan, and G. Hoatson, *Magn. Reson. Chem*., 2002, **40**, 70–76.

2. J. C. Jansen, F. J. van der Gaag and H. van Bekkum. *Zeolites*, 1984, **4**, 369–372.

3. F. Fan, K. Sun, Z. Feng, H. Xia, B. Han, Y. Lian, P. Ying and C. Li, *Chem. Eur. J*., 2009, **15**, 3268–3276.

4. B. Mihailova, M. Wagner, S. Mintova and T. Bein, *Stud. Surf. Sci. Catal*., 2004, **154**,163–170.
